# Hospital acquired pneumonia risk factors in children with Acute Lymphoblastic Leukemia on chemotherapy

**DOI:** 10.1016/j.heliyon.2021.e07209

**Published:** 2021-06-06

**Authors:** Anitha Marllyin Mairuhu, Mia Ratwita Andarsini, Retno Asih Setyoningrum, Andi Cahyadi, Maria Christina Shanty Larasati, I.Dewa Gede Ugrasena, Bambang Permono, Satrio Budiman

**Affiliations:** Department of Child Health, Universitas Airlangga, Faculty of Medicine Dr. Soetomo General Academic Hospital, Surabaya Indonesia

**Keywords:** Hospital-acquired pneumonia, Acute lymphoblastic leukemia, Chemotherapy, Neutropenia, Leukemia, Pneumonia

## Abstract

**Background:**

Over the past 10 years, infection has remained as the main cause of illness and mortality among children with Acute Lymphoblastic Leukemia on chemotherapy. The high incidence of Hospital-Acquired Pneumonia in children with Acute Lymphoblastic Leukemia on chemotherapy with risk factors should be intervened earlier

**Methods:**

An observational case control study of children with Acute Lymphoblastic Leukemia on chemotherapy. Patient with Hospital-Acquired Pneumonia considered as case and patient without Hospital-Acquired Pneumonia as control to analyze risk factors that affect the incidence of Hospital-Acquired Pneumonia in children with Acute Lymphoblastic Leukemia on chemotherapy from 2016 to 2018 was performed in the pediatric ward Dr. Soetomo General Academic Hospital with a total sampling technique. Nine risk factors were analyzed: age, gender, nutritional status, length of stay, risk stratification, chemotherapy phase, anemia, neutropenia, and thrombocytopenia. Bivariate and multivariate analysis using chi-square, continuity correction, and logistic regression was used for statistical analysis.

**Results:**

120 children enrolled the study. Analyzed of risk factors showed risk stratification (p = 0.009), chemotherapy phase (p < 0.001), and neutropenia (p < 0.001) was proven to significantly affect the incidence of Hospital-Acquired Pneumonia in children with Acute Lymphoblastic Leukemia on chemotherapy. Age, gender, nutritional status, length of stay, anemia, and thrombocytopenia were not proven to be a risk factor that affects the incidence of Hospital-Acquired Pneumonia in children with Acute Lymphoblastic Leukemia on chemotherapy.

**Conclusion:**

The incidence of Hospital-Acquired Pneumonia in children with Acute Lymphoblastic Leukemia on chemotherapy is significantly affected by the risk stratification, chemotherapy phase, and neutropenia.

## 1 Introduction

In the last decades, infection has always been the cause of morbidity and mortality on pediatric patient with ALL on chemotherapy [1]. Lungs are often found as the most common source of single infection during chemotherapy. Of all the complications of infections that arise, HAP is the most common complication, ranging from 13 to 31 percent of patient undergoing induction phase of chemotherapy for leukemia [2]. Chemotherapy intensity, neutropenia, Down syndrome patients, and female gender are associated with a higher risk of infection-related deaths [3, 4, 5].

Hospital-Acquired Pneumonia is one of the infection-related complications for high-risk patients with ALL who experience neutropenia [2]. In addition to the effects of neutropenia-induced chemotherapy, disorders of the immune system associated with underlying cancer can increase the risk of infection and lead to premature mortality [6]. A study conducted in Texas showed that the duration of hospital stay was also one of the risks of developing HAP in ALL patients with chemotherapy with an average hospitalization of 13.4 days [7]. Besides, HAP is also one of the most serious infectious complications, with a case fatality rate of 25–45% [6, 7]. A study in Denmark from 1992 to 2001 regarding the incidence of infection in children with ALL during chemotherapy which caused death was found to be highest in patients with high-risk stratification by 57% with p < 0.001 [5].

Premature mortality during the induction phase of chemotherapy in patients with ALL continues to increase significantly despite advances in the quality of supportive care. In part, premature death was due to infectious complications that arose during periods of prolonged chemotherapy and prolonged hospital stay [7]. The frequency of treatment-related mortality in all contemporary trials was reported to be 2–4%, mostly due to infection. Minor modifications in the intensive conventional induction regimen can lead to higher infection-related morbidity and mortality [1]. The high incidence of HAP with risk factors that should have been intervened earlier is the background for researching risk factors that influence the incidence of HAP in ALL children in chemotherapy. By knowing these risk factors, it is hoped that health workers can quickly and accurately identify cases of HAP, as well as provide management and evaluation of HAP to reduce mortality and morbidity due to HAP in ALL children in chemotherapy.

## 2 Materials and methods

This study is an observational study with a case-control study design to analyze the factors that influence the incidence of HAP in ALL children in chemotherapy. This study was granted ethical approval by the Ethic Committee of Dr. Soetomo General Academic Hospital No. 0981/KEPK/II/2019 on February 27^th^, 2019. The sample in this study consisted of the case group and the control group. The case group consists of children aged 0–18 years with ALL on chemotherapy and diagnosed with HAP based on WHO criteria who were treated in the Pediatric Ward Dr. Soetomo General Academic Hospital Surabaya. The control group consists of children aged 0–18 years with ALL on chemotherapy who did not have HAP. All patients received the same chemotherapy protocol, Indonesian ALL-2013 Protocol, according to the risk stratification. The sample size of the case group in this study was 60 patients and the control group sample was taken randomly with a ratio of 1:1. Primary data of the study was taken from medical record data for 3 years from January 1, 2016, to December 31, 2018. Nine risk factors were analyzed: age, gender, nutritional status, length of stay, risk stratification, chemotherapy phase, anemia, neutropenia, and thrombocytopenia.

The baseline data from the history, physical, and radiological examinations were written in a special data collection sheet. The data was sorted and coded according to the research plan for further processing. Subjects with incomplete data were excluded from this study. Data were entered in the Statistical Software Program for Social Science (SPSS). Bivariate and multivariate analysis was used to see the relationship between the independent and dependent variables using the chi-square test, continuity correction, and logistic regression. Other statistical tests were used as needed.

## 3 Results and discussion

The total number of research subjects included in this study were 120 children. Most of the subjects were less than 1 year of age and more than 10 years of age and were male. Subjects with poor nutritional status and malnutrition amounted to 63.3%. As many as 55.8% of subjects were hospitalized for more than 14 days. The research subjects with high-risk stratification amounted to 45%. A total of 53.3% of subjects were undergoing induction phase chemotherapy. The subjects of the study were 43.3% with anemia, 39.2% with neutropenia, and 48.3% with thrombocytopenia. The characteristics of the research subjects are described in Table 1.

**Table 1:** Patients Characteristics and HAP risk factors bivariate analysis.

Variable	Case n (%)	Control n (%)	OR	95% CI	*p-*value
**Age**					
• <1 year of age &>10 years of age	42 (70)	31 (51.7)	2.18	1.03–4.61	0.061
• 1–10 years of age	18 (30)	29 (48.3)			
**Gender**					
• Male	36 (60)	38 (63.3)	1.15	0.55–2.40	0.851
• Female	24 (40)	22 (36.7)			
**Nutritional Status**					
• Poor nutritional status	43 (71,7)	33 (55)	2.07	0.97–4.41	0.088
• Good nutritional status	17 (28,3)	27 (45)			
**Length of Stay**					
• ≥14 days	40 (66,7)	27 (45)	2.44	1.16–5.12	0.027
• <14 days	20 (33,3)	33 (55)			
**Risk Stratification**					
• High risk	33 (55)	21 (35)	2.27	1.08–4.73	0.044
• Standard risk	27 (45)	39 (65)			
**Chemotherapy Phase**					
• Phase I (Induction)	48 (80)	16 (26.7)	11	4.68–25.81	<0.001
• Phase II, Phase III, or Final phase (consolidation, intensification, maintenance)	12 (20)	44 (73.3)			
**Anemia**					
• Hb < 10 g/dL	29 (48,3)	23 (38.3)	1.50	0.72–3.11	0.357
• Hb ≥ 10 g/dL	31 (51,7)	37 (61.7)			
**Neutropenia**					
• ANC <500/μL	45 (75)	2 (3.3)	87	18.91–400.14	<0.001
• ANC ≥500/μL	15 (25)	58 (96.7)			
**Thrombocytopenia**					
• Platelets <50000/μL	34 (56.7)	24 (40)	1.96	0.94–4.05	0.100
• Platelets ≥50000/μL	26 (43.3)	36 (60)			

Bivariate analysis using chi-square and continuity correction was used to analyze the effects of age, gender, nutritional status, duration of hospital stay, risk stratification of ALL, chemotherapy phase, anemia, neutropenia, and thrombocytopenia risk factors on the incidence of HAP in children with ALL. The results of the analysis are presented in Table 1.

Of the 9 risk factors analyzed, duration of hospital stay (p = 0.02), risk stratification (p = 0.04), chemotherapy phase (p < 0.01), and neutropenia (p < 0.01) were shown to significantly affect the incidence of HAP in children with ALL in chemotherapy. Meanwhile, age, gender, nutritional status, anemia, and thrombocytopenia were not proven to influence the incidence of HAP in children with ALL on chemotherapy.

In Table 1, there was no association between age and HAP in children with ALL on chemotherapy based on the analysis of the chi-square test and continuity correction with a p-value = 0.061 and 95% CI 1.03–4.61. However, quantitatively, in the <1 year of age and >10 years of age groups, more patients had HAP (n = 42) than those who did not (n = 31). Inaba *et al.* [1] compared the effect of age on the state of infection-related complications during treatment in pediatric patients with ALL over or equal to 10 years of age compared with patients 1–9.9 years of age and concluded that there was a significant association between age and the risk of infection during the induction chemotherapy phase with p < 0.01. A study in China showed that patients which are less than one year of age are a risk factor for respiratory tract morbidity. This may be due to the fact that the immune system is not well developed, the respiratory tract is still narrow, the bronchial tree is relatively short and the lung development of the child is not yet complete [8]. The difference in the results of the study could be due to the fact that in this study there were no samples with <1 year of age. Age is one of the prognostic factors in ALL, the age limit under 1 year and/or above 10 years is associated with a positive Minimal Residual Disease (MRD) outcome and is associated with gene fusion disorders. MRD is a term that refers to the number of leukemia cancer cells remaining in the body after giving therapy. At the age of ≥10 years, BCR-ABL gene fusion is found and at the age of 1–10 years the TEL-AML1 gene fusion is found. BCR-ABL gene fusion has a poor prognosis concerning chemotherapy resistance which is related to infection risk [9].

There was no association between gender and HAP in children with ALL in chemotherapy based on the chi-square test analysis and continuity correction with p-value = 0.851 and 95% CI 0.55–2.40. However, quantitatively, in the female sex group, more patients had HAP (n = 24) than those who did not (n = 22). There are several differences in the results of studies regarding gender as a risk factor for infection. According to Christensen *et al.* [5], females had a higher risk of infection during ALL treatment than males with p < 0.001. While in the study conducted by Sano *et al.* [10], it was found that there was no difference in risk between pediatric and adolescent patients with hematologic disease and infectious malignancies and without sex-related infections. Differences in research results could be due to differences in research methods. Differences can also be caused by differences in the number and characteristics of the study sample, in which the research was conducted Christensen *et al.* [5] has research subjects as many as 1652 children and the study was conducted for 9 years. Until now, it has not been known with certainty the relationship between sex and the risk of incidence of HAP in ALL children in chemotherapy, therefore it needs to be confirmed in a wider study.

There was no association between nutritional status and HAP in children with ALL on chemotherapy based on the chi-square test analysis and continuity correction with p-value = 0.088 and 95% CI 0.97–4.41. However, quantitatively, in the malnutrition group, more patients had HAP (n = 43) than those who did not (n = 33). Malnutrition can lead to impairment of the immune responses because of decreased production of complement, cytokines, and immunoglobulins due to malnutrition [11]. The nutritional status is often impaired by the natural progression of malignant diseases. Protein-calorie malnutrition that comes from inadequate intake of carbohydrates, protein, and fat to meet metabolic needs and/or reduced macronutrient uptake is the most common secondary diagnosis in individuals diagnosed with cancer. Deficiencies in these nutrients are associated with an increased risk of infection [12, 13]. Nutritional deficiencies can also affect the ability of normal tissues to withstand the toxicity of chemotherapy resulting in the need to discontinue or reduce chemotherapy doses. However, the impact on leukemia-related infection is not well defined [3]. Dissociation between nutritional status and HAP in children with ALL on chemotherapy could be caused by differences in research methods, differences in the number, and differences in the characteristics of the research sample.

Length of stay was a risk factor affecting the incidence of HAP in children with ALL on chemotherapy based on the chi-square test analysis and continuity correction with a p-value = 0.027 and 95% CI 1.16–5.12. The effect of duration of hospital stay is weak (contingency coefficient 0.21), patients with a duration of hospital stay ≥14 days have 2.4 times risk of developing HAP compared to patients with duration of hospital stay <14 days (OR = 2.4). A retrospective cohort study in Texas showed that pneumonia in patients with ALL after the induction phase of chemotherapy continues to be a major cause of problems associated with significant morbidity, mortality, and use of resources. In terms of use of health resources, pneumonia was associated with an increase in the duration of stay in the hospital, where the cumulative incidence of pneumonia was 21% with a duration of hospital stay of 15–34 days, p < 0.001 [7]. In this study and other studies, it is shown that the duration of hospital stay is a risk factor for developing HAP in children with ALL on chemotherapy.

The risk stratification of ALL was a risk factor affecting the incidence of HAP in children with ALL on chemotherapy based on the chi-square test analysis and continuity correction with a p-value = 0.044 and 95% CI 1.08–4.73. The effect of risk stratification of ALL is weak (contingency coefficient 0.19), patients with high-risk stratification have 2.2 times the risk of developing HAP compared to patients with low-risk stratification (OR = 2.2). Inaba *et al.* [1], comparing the effect of risk stratification of ALL on the state of infection-related complications during chemotherapy revealed that high-risk stratification was significantly associated with an increased risk of infection-related complications with p < 0.001. A study in Denmark from 1992 to 2001 regarding the incidence of infection in children with ALL during chemotherapy which caused death was found to be highest in patients with high-risk stratification by 57% with p < 0.001 [5]. This study and several other studies showed that risk stratification is a risk factor for developing HAP in children with ALL on chemotherapy.

The chemotherapy phase was a risk factor affecting the incidence of HAP in children with ALL on chemotherapy based on the chi-square test analysis and continuity correction with p-value <0.001 and 95% CI 4.68–25.81. The effect of this chemotherapy phase is strong (contingency coefficient 0.51). Patients with the phase I (induction) of chemotherapy had 11 times the risk of developing HAP compared to patients with phase II, phase III, or final phase (consolidation, intensification, maintenance) (OR = 11). A study conducted in Canada with a study period from 1997 to 2006 found that most infections occurred during the induction phase of chemotherapy. Among a total of 425 children who were the subjects of the study, 353 children (83.1%) experienced infections in the induction phase of chemotherapy [3]. This study and several other studies showed that the chemotherapy phase was a risk factor for the occurrence of HAP in children with ALL on chemotherapy.

There was no association between anemia and HAP in children with ALL on chemotherapy based on the chi-square test analysis and continuity correction with p-value = 0.357 and 95% CI 0.72–3.11. However, quantitatively, in the Hb < 10g/dL group more patients had HAP (n = 29) than those who did not (n = 23). A study by Garcia *et al.* [7] about pneumonia in patients with ALL after the induction phase of chemotherapy showed that there was no significant relationship between anemia and the incidence of pneumonia. The highest risk of infection occurred during day 10 to day 20 according to the time of most frequent transfusions. Transfusions are known to cause lung injury and affect the immune response, which contributes to the incidence of pneumonia [7]. This study and other studies indicate that anemia is not a risk factor for developing HAP in children with ALL on chemotherapy. Pizzo *et al.* argued that the emergence of anemia in patients with ALL is due to the bone marrow losing its function to produce red blood cells. Loss of bone marrow function is due to the progressive infiltration of white blood cells into the bone marrow so that the bone marrow cannot produce red blood cells properly. Anemia with a Hb < 10 g/dL occurs in about 80% of patients at the time of diagnosis [14].

Neutropenia was a risk factor affecting the incidence of HAP in children with ALL in chemotherapy based on the chi-square test analysis and continuity correction with p-value <0.001 and 95% CI 18.91–400.14. The effect of this neutropenia was strong (contingency coefficient 0.59) and patients with neutropenia had an 87 times risk of developing HAP compared to patients with low-risk stratification (OR = 87). Bodey *et al.* [15] described the inverse relationship between absolute neutrophil count and infection risk. Since then, chemotherapy-induced neutropenia has been the most widely recognized risk factor for cancer-related bacterial pneumonia. Neutrophils are very sensitive to nucleoside analogues and alkylating agents, both of which cause a reduction in the dose depending on the level of circulating neutrophils. Severe neutropenia (<500 cells/μl) is associated primarily with serious lung infections and poor outcome. Underlining the relevance of these risk factors, Vento *et al.* [6] estimates that nearly 60% of cancer patients who develop chemotherapy-induced neutropenia will develop lung infiltrates on radiographs. The rate of onset, duration, severity, and physiological processes underlies all susceptibility which has further impacts on neutropenic pneumonia [6]. Besides, impaired neutrophil phagocytosis, and chemotaxis follow common cancer-related elimination processes such as radiation, corticosteroids, hypovolemia, acidosis, and hyperglycemia. Thus, functional neutropenia may also contribute to the risk of cancer-related pneumonia [16]. In this study and several other studies, it was shown that neutropenia was a risk factor for developing HAP in children with ALL in chemotherapy.

There was no association between thrombocytopenia and HAP in children with ALL in chemotherapy based on the chi-square test analysis and continuity correction with p-value = 0.100 and 95% CI 0.94–4.05. However, quantitatively, in the platelet group <50000/μL, more patients had HAP (n = 34) than those who did not (n = 24). A retrospective cohort study in Texas with a study sample taken from 2005 to 2009 showed that there was no relationship between thrombocytopenia and the incidence of pneumonia in patients with ALL after the induction phase of chemotherapy. The state of thrombocytopenia early in the disease is only a marker for deeper bone marrow involvement by leukemia [7]. Research by Li *et al.* [17] shows that platelets have an important function in inflammation and immune response, so further studies are needed to confirm the relationship of thrombocytopenia with the incidence of HAP. In this study and other studies, it was shown that thrombocytopenia was not a risk factor for developing HAP in children with ALL on chemotherapy. Until now, there is no definite relationship between thrombocytopenia and the risk of HAP in children ALL on chemotherapy, therefore it needs to be confirmed with a wider study.

The multivariate analysis aims to look at the risk factors that have the greatest influence on the incidence of HAP in children with ALL on chemotherapy. The analysis conducted in this study is logistic regression analysis with the simultaneous method. The results of the analysis can be seen in Table 2. Of the 9 factors that influence the incidence of HAP in children with ALL on chemotherapy, a logistic regression test was carried out for age, nutritional status, duration of hospital stay, risk stratification of ALL, chemotherapy phase, neutropenia, and thrombocytopenia. In this multivariate analysis, it was found that the risk factors for length of stay were not consistent enough to influence the incidence of HAP in children with ALL on chemotherapy. From the analysis, only risk factors for ALL risk stratification, chemotherapy phase, and neutropenia had a p-value <0.05.

**Table 2:** HAP risk factors multivariate analysis.

Variable	*Adjusted* OR	95% CI	*p-value*
Age	3.87	0.77–19.39	0.100
Nutritional status	4.78	0.79–28.66	0.087
Length of stay	0.75	0.14–3.82	0.734
Risk stratification	21.58	2.17–214.18	0.009
Chemotherapy phase	43.88	5.29–363.50	<0.001
Neutropenia	479.93	32.87–7005.81	<0.001
Trombocytopenia	0.23	0.02–2.14	0.235

The limitation of this study is that matching is carried out for the selection of control samples. Further studies with prospective research methods are needed to prove the risk factors that can influence the incidence of HAP in children with ALL on chemotherapy.

## 4 Conclusion

The incidence of HAP in children ALL on chemotherapy was significantly influenced by risk factors risk stratification of ALL, chemotherapy phase, and neutropenia. Risk factors for age, sex, nutritional status, length of stay, anemia, and thrombocytopenia were not proven risk factors for the incidence of HAP in ALL children on chemotherapy. This study was the first study that analyze risk factor that influence the incidence of HAP in ALL children in chemotherapy. However, further study with greater sample size and matching of the control-sample selection is needed.

## Declarations

### Author contribution statement

Anitha Marllyin Mairuhu, Mia Ratwita Andarsini, Retno Asih Setyoningrum, Andi Cahyadi, Satrio Budiman, Maria Christina Shanty Larasati, I Dewa Gede Ugrasena and Bambang Permono: Conceived and designed the experiments; Analyzed and interpreted the data; Contributed reagents, materials, analysis tools or data; Wrote the paper.

### Funding statement

This research did not receive any specific grant from funding agencies in the public, commercial, or not-for-profit sectors.

### Data availability statement

Data included in article/supplementary material/referenced in article.

### Declaration of interests statement

The authors declare no conflict of interest.

### Additional information

No additional information is available for this paper.
